# Mutation analysis using cell-free DNA for endocrine therapy in patients with HR+ metastatic breast cancer

**DOI:** 10.1038/s41598-021-84999-9

**Published:** 2021-03-10

**Authors:** Sung Hoon Sim, Han Na Yang, Su Yeon Jeon, Keun Seok Lee, In Hae Park

**Affiliations:** 1 Translational Cancer Research Branch, Research Institute, National Cancer Center, Goyang, Republic of Korea; 2 Center for Breast Cancer, National Cancer Center Hospital, National Cancer Center, Goyang, Republic of Korea; 3 Present Address: Division of Hematology/Oncology, Department of Internal Medicine, Korea University College of Medicine, Guro Hospital, 148, Gurodongro, Gurogu, Seoul, 08308 Republic of Korea

**Keywords:** Cancer, Genetics

## Abstract

We prospectively evaluated the utility of *ESR1* and *PIK3CA* mutation analysis with cell-free DNA (cfDNA) using droplet digital PCR (ddPCR) for the efficacy of endocrine therapy (ET) in hormone receptive positive (HR+) metastatic breast cancer (MBC) patients. CfDNA was analyzed just before the start of ET for MBC. *E380Q, Y537N, Y537S,* and *D538G* were assessed for *ESR1* mutations and *H1047R, E545K*, and *E542K* were assessed for *PIK3CA* mutations. A total of 75 patients were enrolled. Of those, 31 (41.3%) received letrozole with palbociclib, and 28 (37.3%) received exemestane and everolimus (EverX). *ESR1* mutations were found in 36 (48.0%) patients, of which 16 (21.3%) had more than one variant. Seventeen (23.6%) patients had one *PIK3CA* mutation and 8 (11.1%) had two. In the total population, time to progression of the first ET after enrollment (TTP1) decreased significantly as the number of *ESR1* mutations increased (*p* < 0.001). *PIK3CA* mutations were also significantly associated with shorter TTP1 (median TTP1: 16.2 months vs. 10.9 months, *p* = 0.03). In contrast, *PIK3CA* mutations were significantly associated with longer TTP in patients receiving EverX treatment (median TTP of EverX: 15.9 months vs. 5.2 months, *p* = 0.01) and remained a significant factor in multivariable analysis for TTP of EverX in this subgroup (hazard ratio = 0.2, 95% CI = 0.1– 0.8, *p* = 0.03). *ESR1* and *PIK3CA* mutations in cfDNA were associated with clinical efficacies of ET in HR+ MBC patients.

## Introduction

Gaining knowledge of genetic variants of patients by molecular testing can help the management of personalized therapies. There is considerable evidence indicating the difference between primary tumor characteristics and those occurring during metastases^1–4^. In addition, high levels of intratumoral or intertumoral heterogeneity may confer tumor treatment resistance and lead to poor clinical outcomes^5^. Intratumoral or intertumoral heterogeneity can be distinguished by spatial and temporal heterogeneities, which involve dynamic variations in the genetic diversity of individual tumors over time^3,5^. In the real world, it is difficult to conduct tumor biopsies on a regular basis, so most therapeutic agents are selected based on the status of the primary tumor. However, serial characterization of genetic variants by liquid biopsy may be useful because of its potential to provide valuable information about tumor heterogeneity^6^. Liquid biopsies, particularly those involving cell-free DNA (cfDNA) from plasma, are emerging as an important alternative approach for current means of molecular profiling of tumors^6–8^. Analysis using cfDNA has potential to make it possible to detect tumors in their early stages^9^, monitor tumor recurrence^10^, and predict the sensitivity of treatments^11^.

The estrogen receptor (ER) is a nuclear hormone receptor that is expressed in approximately 60–70% of breast cancers^12^. The ERα is encoded by *ESR1* and is a main target of endocrine therapy (ET), which is widely used for both early and metastatic hormone receptor (HR) positive breast cancer treatments^13^. Mutations of *ESR1* have been reported in approximately 20–30% of patients treated by aromatase inhibitors (AIs) or tamoxifen and are known to be acquired mutations, which confer resistance to ET^14^. Most *ESR1* mutations are found in the ligand-binding domain of ERα, which is assumed to retain sensitivity to other endocrine agents, such as fulvestrant, which cause ERα degradation^15,16^.

Like *ESR1*, *PIK3CA* is a promising predictive biomarker for HR+ breast cancer in patients treated with targeted therapy for the PI3K pathway. Approximately 40% of HR+/HER2− breast cancers are known to contain activating mutations in *PIK3CA*, which lead to decreased ET efficacy^17^. Two targeted therapies for the PI3K pathway are actively used with ET for HR+ metastatic breast cancers (MBCs); namely, the mammalian target of rapamycin (mTOR) inhibitor (everolimus) and a PI3Kα-specific inhibitor (alpelisib)^18,19^. Several retrospective studies based on clinical trials with ET and/or targeted therapy have reported that *ESR1* or *PIK3CA* mutations are associated with shorter progression-free survival and overall survival^19–24^, though the magnitudes of risks or benefits differ according to the study population and therapeutic regimens used.

Considering the rapid development of endocrine and targeted therapies, knowing the genetic profiles of HR+ breast cancer patients before selecting the drug treatment is increasingly important for patient care. However, there have been few prospective biomarker studies so far other than trial-based retrospective adjunctive studies. In addition, the appropriate diagnostic method for clinical application has not yet been determined. The various targeted gene sequencing panels on the market are too expensive to be tested sequentially with the continuation of treatment. In this study, therefore, we prospectively evaluated the clinical availability and utility of *ESR1* and *PIK3CA* mutation analysis in cfDNA using droplet digital polymerase chain reaction (ddPCR) in a cohort of HR+ metastatic breast cancer (MBC) patients.

## Patients and methods

### Patient population

In this study, patients who were scheduled to receive ET at any stage of the treatment process for their HR+/HER2− MBC were enrolled. Major inclusion criteria were as follows: confirmed HR+ and HER2− MBC, Eastern Cooperative Oncology Group (ECOG) scale of performance status of 0 to 2, no symptomatic visceral involvement, and life expectancy of more than 12 months. From December 2016 to December 2018, a total of 107 patients were enrolled and 32 patients were excluded for the following reasons: adjuvant treatment setting (n = 11), HER2+ disease (n = 5), and no further ET (n = 16). Among the total patient group, patients who received exemestane and everolimus combination therapy were classified as the EverX group; the correlation between *PIK3CA* mutation and clinical results was analyzed separately for this group.

cfDNA was analyzed just before the start of ET following the diagnosis of MBC or disease progression during prior therapy. Clinical decisions were made regardless of the results of cfDNA analysis, and all data were blinded until the last analysis. This study was approved by the Institutional Ethical Committee of the National Cancer Center, Republic of Korea. Written informed consent was obtained from all patients before drawing blood.

### DNA extraction from plasma

6 ml of blood collected in EDTA blood collection tubes was centrifuged at 1600*g* for 20 min to separate the plasma. Plasma was aliquoted and stored at − 70 °C until DNA extraction. DNA was isolated from 1 mL of plasma using the MagMax Cell-Free DNA Isolation Kit (Thermo Fisher, Logan, UT, USA). Isolated plasma DNA was quantified and qualified using Qubit dsDNA HS (High Sensitivity) Assay Kit (Invitrogen cat #Q32851).

### *ESR1* and *PIK3CA* mutations detection by Droplet Digital PCR (ddPCR)

The Bio-Rad QX100 ddPCR platform (Bio-Rad, Hercules, CA, USA) was used for sensitive detection of *ESR1* and *PIK3CA* mutations. Primers and probes were designed for *ESR1* p.E380Q, p.Y537S, p.Y537N, and p.D538G, as described in a prior study (Table S1)^23^. *PIK3CA* mutations were detected using customized assays for *PIK3CA H1047R* (dHsaCP2000077), *E542K* (dHsaCP2000073), and *E545K* (dHsaCP2000075) (Bio-Rad, Hercules, CA, USA) on a Bio-Rad QX100 Droplet Digital PCR System.

The samples were prepared by mixing 10 µL of ddPCR Supermix for probes (no deoxyuridine triphosphate [dUTP], Bio-Rad) and 2 µL from the ddPCR probe assay kit (Bio-Rad, and Thermo Fisher Scientific), which consisted of forward and reverse PCR primers, a FAM or VIC-labeled fluorescent probe specific for mutant or wild type, and 8 µL of template DNA in a final reaction volume of 20 µL. Droplets were generated by a QX100 droplet generator (Bio-Rad) with 70 µL droplet generation oil and 20 µL reaction volume. Droplets in each sample were transferred into a PCR plate for amplification followed by the thermal cycling program; incubation at 95 °C for 10 min, 40 cycles of 94 °C for 30 s, 55 °C for 60 s, and holding at 98 °C for 10 min. After the amplification was done, the plates were transferred to a QX100 Droplet reader (Bio-Rad). Each assay run included wild type normal human DNA (TaqMan control Genomic DNA) and no template (water only) for negative controls. All assays were reviewed to evaluate amplitude threshold and clear separation of positive and negative partitions. A mutation was determined positive with more than one FAM–positive (mutation positive) droplets were detected. A fractional abundance of mutant DNA alleles to total DNA alleles was calculated from the number of FAM-positive events over total positive events (both FAM and VIC positive for mutation and wild type, respectively) using the Quanta Soft analytical software package (Bio-Rad). All of these experiments were referenced to the manufacturer’s guide (Droplet Digital PCR Application Guide, Bio-Rad, http://www.bio-rad.com/ddPCRAppGuide) and performed in duplicate.

### Statistical analysis

Baseline characteristics of patients were compared by Chi-squared and Wilcoxon rank sum tests. Time to progression (TTP) was defined as the length of time from the start of the ET until disease progression; TTP1 was defined as the time to progression of the first ET; and TTP of exemestane and everolimus (EverX) treatment was defined as the same meaning as the EverX treatment that was administered as the first or second ET during the study period. The median TTP1 and TTP of EverX were estimated using the Kaplan–Meier method and compared using the log-rank test. Multivariable analysis was performed using the Cox proportional hazards model to assess the effect of mutant forms against the wild-type, with adjustments for clinical factors of prior endocrine therapy, prior chemotherapy or visceral metastasis. Values of *p* < 0.05 were considered statistically significant; all statistical analyses were performed using the SPSS statistical software for Windows, version 21.0 (SPSS, Chicago, IL, USA).

### Ethical approval

All procedures performed in accordance with the Helsinki declaration, Study approval was obtained from the Institutional Review Board at National Cancer Center, Korea before conducting all procedures.

### Informed consent

Written informed consent was obtained from all patients.

## Results

### Patient characteristics

The median age of the study population was 55 years (range 35–87 years) (Table 1). The number of previous hormone therapies at a metastatic setting was zero in 36 patients (48.0%), one in 23 patients (30.7%), two in 13 patients (17.3%), and more than two in three patients (4.0%). The majority of patients did not receive chemotherapy for their MBC (n = 53, 70.7%); however, 13 (17.3%) patients received more than one line of chemotherapy before participating in this study. Of the 75 patients, the median duration of previous AI treatments in the adjuvant and the metastatic setting was 8.0 months (range 0–114 months), and 33 patients (44%) had visceral metastasis at the time of enrollment. All patients received ET after enrollment (the first ET), and 12 (16%) patients received subsequent ET (the second ET) after the failure of the first ET during the study period (Table S2). The letrozole and palbociclib combination (n = 31, 41.3%) was most commonly used as the first ET. Exemestane and everolimus (EverX) was used as the first ET in 19 (25.3%) patients and as the second ET in nine patients (12%).

**Table 1:** Patient characteristics.

	Total (n = 75)	*ESR1* genotype (n = 75)		*PIK3CA* genotype (n = 72^‡^)	
		*ESR1*m+ (n = 36)	Wild (n = 39)	*PIK3CA*m+ (n = 25)	Wild (n = 47)
Age (years, median, range)	55 (35–87)	53 (35–79)	58 (38–87)	56 (35–87)	54 (38–72)
		*p* = 0.13		*p* = 0.62	
Neo/Adjuvant ET*					
None	30 (40.0%)	14 (38.9%)	16 (41.0%)	11 (44.0%)	17 (36.2%)
Tamoxifen	33 (44.0%)	17 (47.2%)	16 (41.0%)	10 (40.0%)	22 (46.8%)
AI^†^	12 (16.0%)	5 (13.9%)	7 (17.9%)	4 (16.0%)	8 (17.0%)
		*p* = 0.83		*p* = 0.80	
Previous ET for MBC**					
0	36 (48.0%)	13 (36.1%)	23 (59.0%)	9 (36.0%)	27 (57.4%)
1	23 (30.7%)	15 (41.7%)	8 (20.5%)	10 (40.0%)	12 (25.5%)
2	13 (17.3%)	6 (16.7%)	7 (17.9%)	6 (24.0%)	6 (12.8%)
≥ 3	3 (4.0%)	2 (5.6%)	1 (2.6%)	0	2 (4.2%)
		*p* = 0.05		*p* = 0.08	
Previous CT*** for MBC					
0	53 (70.7%)	20 (55.6%)	33 (84.6%)	16 (64.0%)	36 (76.6%)
1	9 (12.0%)	6 (16.7%)	3 (7.7%)	5 (20.0%)	2 (4.3%)
≥ 2	13 (17.3%)	10 (27.9%)	3 (7.7%)	4 (16.0%)	9 (19.2%)
		*p* = 0.01		*p* = 0.26	
De novo stage IV breast cancer	15 (20.0%)	4 (11.1%)	11 (28.2%)	5 (20.0%)	10 (21.3%)
		*p* = 0.06		*p* = 0.90	
Duration of AI^†^ treatment in the adjuvant and metastatic settings (months, median, range)	8.0 (0–114)	10.7 (0–87)	6.0 (0–114)	17.3 (0–72)	4.9 (0–114)
		*p* = 0.73		*p* = 0.32	
**Metastatic site**					
Bone only	15 (20%)	8 (22.2%)	7 (17.9%)	4 (16.0%)	11 (23.4%)
Visceral	33 (44%)	17 (47.2%)	16 (41.0%)	13 (52.0%)	18 (38.3%)
		*p* = 0.78		*p* = 0.55	

*Endocrine therapy, **metastatic breast cancer, ***chemotherapy, ^†^aromatase inhibitor, ^‡^missing = 3.

### Frequency of *ESR1* and *PIK3CA* mutations

**Figure 1:**
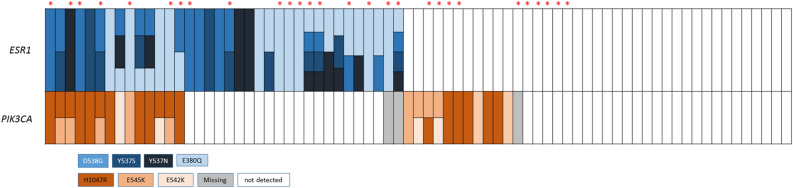
Patient distribution according to *ESR1* and *PIK3CA* hot spot mutations. Mutations are colored according to amino acids. Blank indicates that the mutations assessed were negative and grey indicates data not available. Red stars indicate patients in the EverX group.

The median total cfDNA concentration for the study population was 20.4 ng (range 9.9–157.0 ng). Overall, there was no significant difference in the level of total cfDNA concentration between the patient with and without mutations found in cfDNA (median 20.4 ng vs. 19.5 ng, *p* = 0.87). *ESR1* hot spot (HS) mutations were identified in 36 (48%) patients; the most prevalent was *E380Q* (30.1%), followed by *D538G* (22.7%), *Y537N* (15.7%), and *Y537S* (13.3%) (Table 2). Sixteen patients (21.3%) had more than one *ESR1* HS mutation (Fig. 1). The *PIK3CA* HS mutations were identified in 72 patients and 47 (65.3%) patients had no mutation. The most prevalent *PIK3CA* HS mutations were *H1047R* (22.7%), followed by *E545K* (13.3%) and *E542K* (8.0%) (Table 2). Seventeen patients (23.6%) had one *PIK3CA* HS mutation and eight patients (11.1%) had two mutations. *ESR1* mutations occurred more often in patients who also had *PIK3CA* mutations; that is, 56% of patients with *PIK3CA* mutations also had an *ESR1* mutation compared with 42.6% of patients without a *PIK3CA* mutation (Fig. 1).

**Table 2:** Prevalence of *ESR1* and *PIK3CA* mutations in overall population and EverX subpopulation.

*ESR1* genotype	Overall	EverX
	N = 75	N = 28
Wild type	39 (52.0%)	10 (35.7%)
**Mutation type**	36 (48.0%)	18 (64.3%)
*E380Q*	23 (30.1%)	12 (42.9%)
*D538G*	17 (22.7%)	10 (35.7%)
*Y537S*	10 (13.3%)	5 (17.9%)
*Y537N*	11 (15.7%)	4 (14.3%)
**Number of mutation**		
0	39 (52.0%)	10 (35.7%)
1	20 (26.7%)	11 (39.3%)
2	9 (12.0%)	4 (14.3%)
3	4 (5.3%)	0
4	3 (4.0%)	3 (10.7%)

*PIK3CA* genotype	N = 72	N = 25
Wild type	47 (65.3%)	14 (56.0%)
**Mutation type**	25 (34.7%)	11 (44.0%)
*H1047R*	17 (22.7%)	9 (36.0%)
*E545K*	10 (13.3%)	6 (25.0%)
*E542K*	6 (8.0%)	1 (4.0%)
**Number of mutation**		
0	47 (65.3%)	14 (56.0%)
1	17 (23.6%)	6 (24.0%)
2	8 (11.1%)	5 (20.0%)
3	0	0

There was a notable difference in the frequency of *ESR1* mutations in patients with prior ET (*p* = 0.05), prior chemotherapy (*p* = 0.01), or de novo stage IV breast cancer (*p* = 0.06) (Table 1). One of 4 patients with de novo stage IV breast cancer and *ESR1* mutation harbored two *ESR1* HS mutations (*Y537S* and *E380Q*) and the other three had a single mutation. On the other hand, there was no association between *PIK3CA* mutation prevalence and baseline clinical variables. In the EverX group, more patients had multiple *ESR1* and *PIK3CA* mutations than the total population (Table 2, Fig. 1).

### Correlation between *ESR1/PIK3CA* mutations and endocrine efficacy

**Figure 2:**
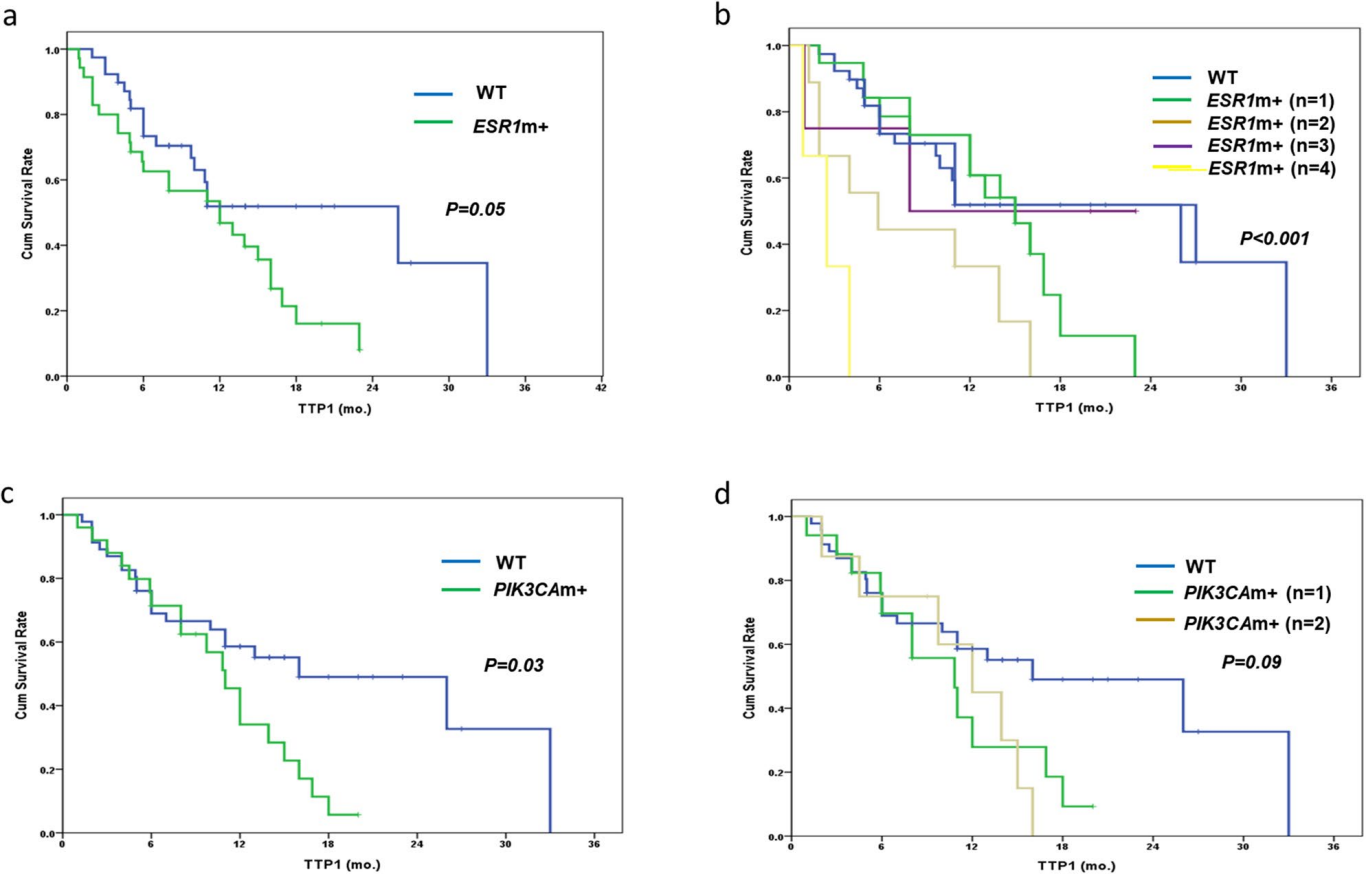
Time to progression after the first ET following enrollment (TTP1) according to the presence of *ESR1* and *PIK3CA* mutations. (**a**) TTP1 according to the presence of *ESR1* mutations. (**b**) TTP1 according to the number of *ESR1* mutations. (**c**) TTP1 according to the presence of *PIK3CA* mutations. (**d**) TTP1 according to the number of *PIK3CA* mutations.

The patients with *ESR1* mutations in their cfDNA showed numerically shorter TTP1 compared to those without *ESR1* mutations [median TTP1: 26.1 months (95% CI 7.5–44.5) vs. 12.0 months (95% CI 5.7–18.3), *p* = 0.05] (Fig. 2a). In addition, there was a clear tendency for shorter median TTP1 as the number of *ESR1* mutations increased (*p* < 0.001) (Fig. 2b). In terms of *PIK3CA*, the presence of mutations was significantly associated with shorter TTP1 in the entire population [median TTP1: 16.2 months (95% CI 5.7–6.3) vs. 10.9 months (95% CI 8.8–13.2), *p* = 0.03]; however, there was no association between the number of mutations and the TTP1 (Fig. 2c,d). The presence of mutations in *ESR1* or *PIK3CA* was an important predictive factor for shorter TTP1, although the values did not reach statistical significance when adjusting for other clinical variables (Table S3).

**Figure 3:**
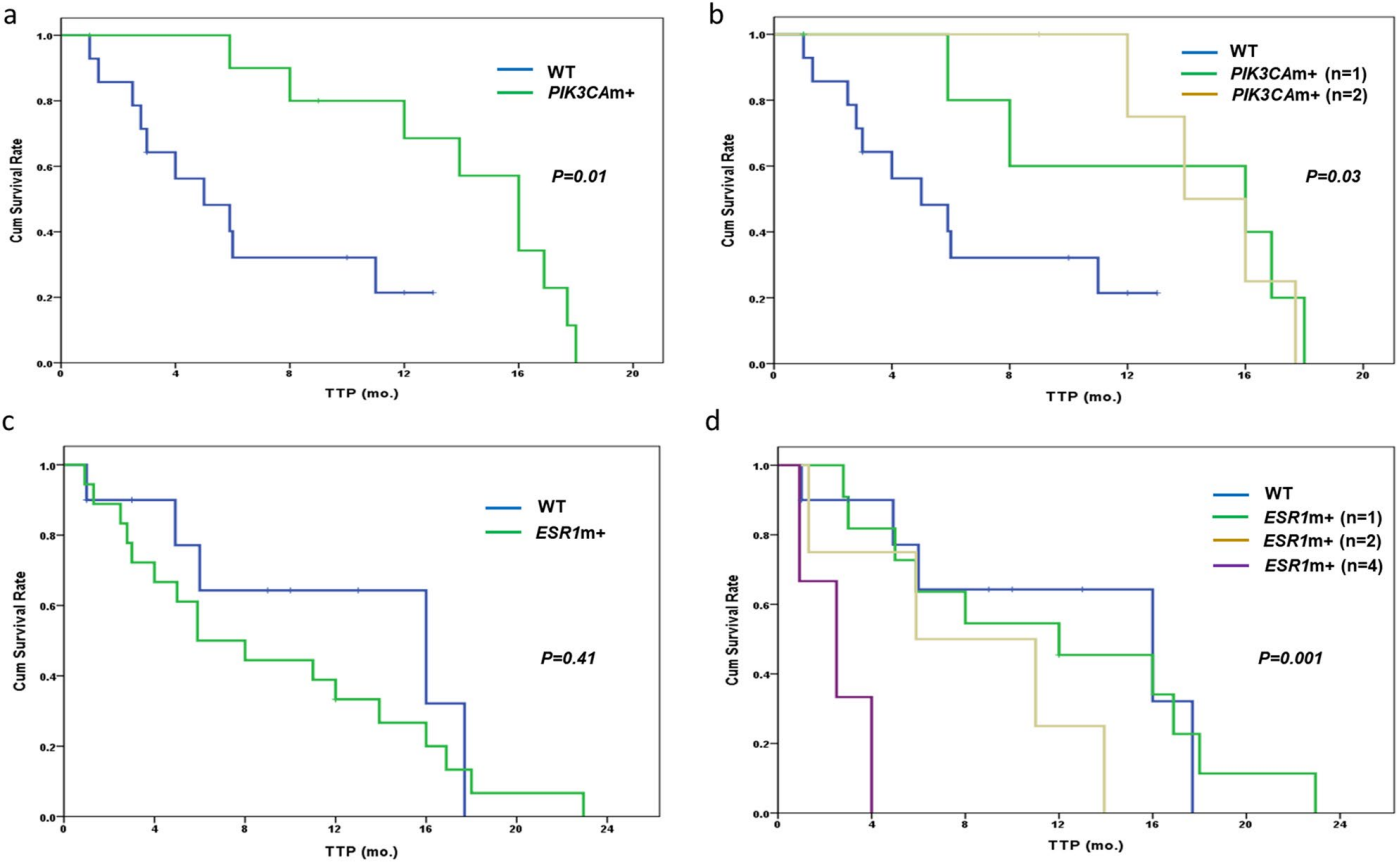
Time to progression (TTP) of EverX (Exemestane+ Everolimus) treatment after enrollment according to the presence of *PIK3CA* and *ESR1* mutations. (**a**) TTP according to the presence of *PIK3CA* mutations. (**b**) TTP according to the number of *PIK3CA* mutations. (**c**) TTP according to the presence of *ESR1* mutations. (**d**) TTP according to the number of *ESR1* mutations.

In contrast, *PIK3CA* mutations were significantly associated with longer TTP for patients receiving EverX treatment [median TTP: 15.9 months (95% CI 13.2–18.8) vs. 5.2 months (95% CI 1.8–8.2), *p* = 0.01] (Fig. 3a). The number of *PIK3CA* mutations was also statistically related to the TTP of EverX treatment (Fig. 3b). In terms of *ESR1*, the number of mutations, not the presence of mutations, correlated with shorter TTP of EverX (Fig. 3c,d). The presence of *PIK3CA* mutations was only a significant prognostic factor for longer TTP in the EverX treatment group (HR = 0.2, 95% CI 0.1–0.8, *p* = 0.03); it also remained an important factor in multivariable analysis with adjustments for clinical factors of prior chemotherapy and visceral metastasis (Table 3).

**Table 3:** Cox analysis for time to progression (TTP) of EverX treatment after enrollment.

	TTP	
	Univariable analysis	Multivariable analysis*
	95% CI, p value	95% CI, p value
Number of prior ET (> 1 vs. 1)	1.7 (0.5–5.9), *p* = 0.43	
Prior CT (yes vs. no)	0.6 (0.2–1.6), *p* = 0.33	0.9 (0.3–2.7), *p* = 0.89
Visceral metastasis (yes vs. no)	0.5 (0.2–1.3), *p* = 0.18	0.8 (0.3–2.4), *p* = 0.73
*ESR1* m+ vs. WT	1.5 (0.6–4.2), *p* = 0.42	
*PIK3CA* m+ vs. WT	0.2 (0.1–0.8), *p* = 0.02	0.2 (0.1–0.8), *p* = 0.03

*EverX* exemestane + everolimus, *CI* confidential interval, *ET* endocrine therapy, *CT* chemotherapy, *WT* wild type. *Adjusted with prior chemotherapy, visceral metastasis, and *PIK3CA* mutation.

**Figure 4:**
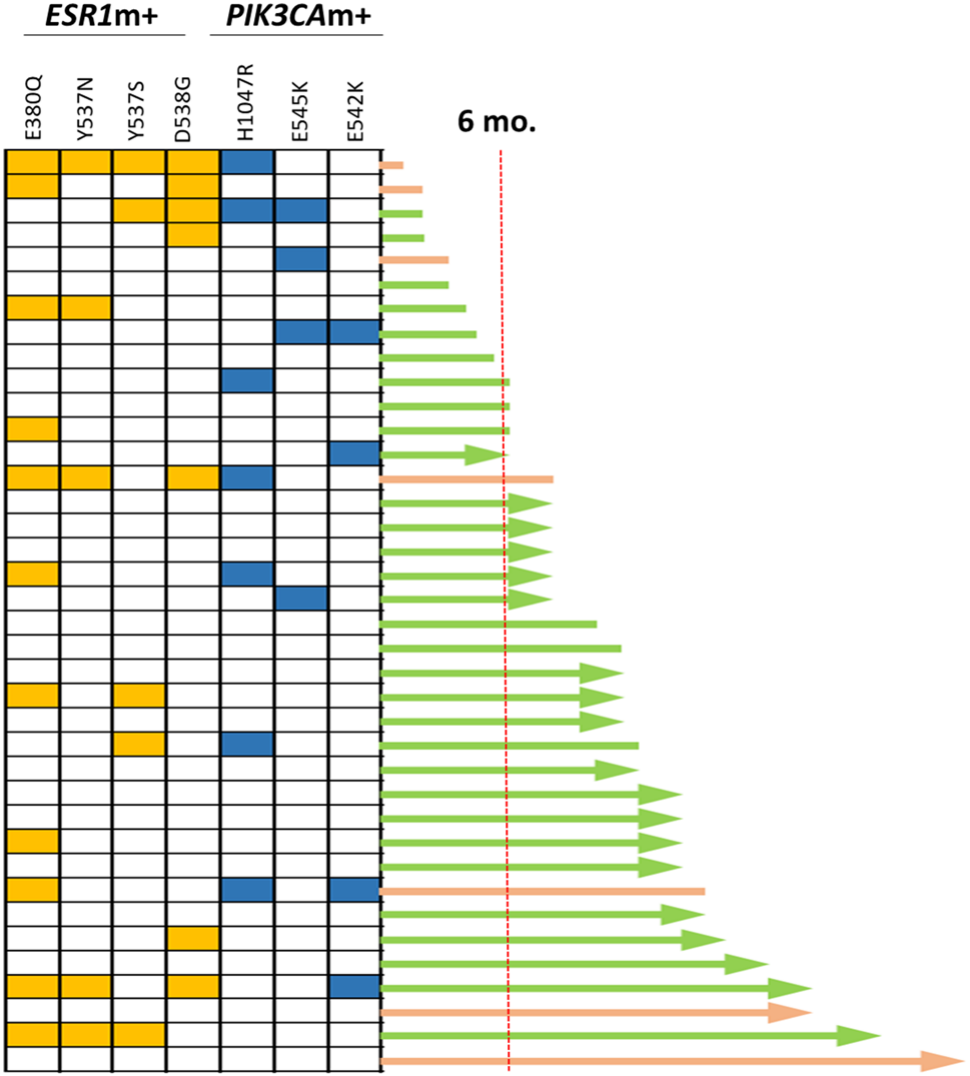
*ESR1* and *PIK3CA* mutation profiles and responses to endocrine therapy with CDK4/6 inhibitors as the first line in 38 patients. Pink line, fulvestrant + CDK4/6 inhibitor; green line, letrozole + CDK4/6 inhibitor; arrow end, ongoing treatment; blunt end, stop due to disease progression; red dotted line, 6 months after the start of treatment; yellow box, *ESR1* mutation; blue box, *PIK3CA* mutation.

In patients receiving ET (letrozole or fulvestrant) with a CDK4/6 inhibitor as the first ET (n = 38), there was a tendency toward a shorter TTP1 being associated with the presence of *ESR1* (HR = 1.9, 95% CI 0.7–5.1, *p* = 0.16) or *PIK3CA* mutations (HR = 2.5, 95% CI 1.0–6.5, *p* = 0.05) (Fig. S1). Of 38 patients, 12 patients experienced disease progression within 6 months after the start of treatment and 9 of those patients (75%) had *ESR1* or *PIK3CA* mutations (Fig. 4). Neither *ESR1* nor *PIK3CA* mutations were associated with overall survival in all patient populations (data not shown).

## Discussion

Endocrine therapy is the main strategy for HR+ breast cancer because of the estrogen-dependent nature of this subtypes. The clinical availability of a number of drugs, like AIs, tamoxifen (selective estrogen receptor modulator, SERM), or fulvestrant (selective estrogen receptor down-regulator, SERD), has led to substantial improvements in survival outcomes for patients with HR+ breast cancer^25,26^. Nevertheless, approximately 20% of patients with early breast cancer experience disease relapse, and most patients with MBC show resistance to treatment^25,27^.

The activation of the PI3K pathway by gain-of-function mutations in *PIK3CA* occurs at a frequency of 30–40% in HR+ breast cancer patients^28^. Among these, *E542K*, *E545K,* and *H1047R* are the most frequently observed HS mutations^28^. *ESR1* mutations are observed mainly in metastatic HR+ breast cancers that have previously been treated with hormone therapy^29^, and all mutations affect the ligand-binding domain of the receptor, especially involving amino acids 537 or 538 in helix 12^29^. These activating mutations are known to endow resistance to hormone therapy. The mTOR1 inhibitor everolimus got the first approval for AI-resistant HR+ MBC as the second line therapy along with ET. Recently, the PI3Kα-specific inhibitor alpelisib has shown promising results for treatment of HR+ MBC harboring *PIK3CA* mutations^18,19^. CDK4/6 inhibitors have shown their efficacies in patients carrying *ESR1* mutations, and a new generation of SERDs are also being tested in clinical trials for these mutations^27^. There are, therefore, several possible combinations or sequences of ET with targeted drugs available for use. With the use of new drugs, there is an increasing need for knowledge of genetic variations to manage therapeutic strategies in HR+ MBC patients. To accomplish this objective, cfDNA analysis may be a possible noninvasive way to obtain serial genetic information in parallel with treatments. However, few studies have evaluated treatment outcomes based only on cfDNA assay-guided targeted therapies prospectively.

In the present study, we conducted cfDNA analyses using the ddPCR method for patients with HR+ MBC before determining treatment options. The ddPCR method is fast, with only 2–3 days of turnaround time for analysis, and is relatively inexpensive. It has a high sensitivity to detect mutant allele fractions (< 0.1%), but can only screen for known variants^30^. We detected activating mutations in *ESR1* and *PIK3CA* in 75 patients, and the overall detection percentages of these mutations were similar to previous results. However, the frequency of *ESR1* mutations in treatment-naïve, de novo stage IV breast cancer patients was significantly higher than that of The Cancer Genome Atlas (TCGA) results that reported that the *ESR1* genomic alteration in primary tumors was < 1%^31^. In addition, the frequency of the *E380Q* variant, previously reported at approximately 9–24%, was 30.1% in this study, which was higher than the frequencies of *D538G* or *Y537S*, known to be the most common mutations^20,32,33^. It was difficult to confirm in this study whether there was a difference in sensitivity to ET due to the high *E380Q* mutation rate or the presence of *ESR1* mutations in patients with de novo stage IV breast cancer. More research is needed to determine whether such discrepancies are due to the differences between metastatic and primary breast cancer or other clinical factors (e.g., age, menopausal status, history of chemotherapy etc.) or differences in analytical methods.

Based on our results, the *PIK3CA* mutation was an especially good predictive factor for the mTOR inhibitor everolimus with ET (EverX treatment). Several preclinical and clinical studies have reported that PI3K pathway activation and *PIK3CA* mutations are related to everolimus efficacy^34,35^. However, in the BOLERO2 study, the benefit of the mTOR1 inhibitor, everolimus was maintained regardless of the presence of *PIK3CA* mutations, which were detected by ddPCR using cfDNA^24^. There might be several reasons why our results are contrary to those of the BOLERO2 study. First, the percentage of patients with multiple mutations was higher in our study than in the BOLERO2 study (20% vs. 0.7%). Second, there is a considerable difference in *PIK3CA* HS mutation profiles between the two study populations. More patients in our EverX treatment group had an *H1047R* mutation in exon 20 than did the patients in the BOLERO2 study (81.8% vs. 56.2%) that could affect the sensitivity to ET and the mTOR inhibitor. Additionally, these disparities could also have originated from the heterogeneous patient population with higher tumor burden and multiple prior treatments in this study.

Currently, the CDK4/6 inhibitor combined with letrozole or fulvestrant is the first choice of therapy for HR+ MBC if patients do not suffer from a visceral crisis. Previously, plasma *ESR1* or *PIK3CA* mutations at baseline were known to not affect the magnitude of the beneficial effect of palbociclib, a CDK4/6 inhibitor; however, these mutations were associated with numerically shorter progression-free survival and lower response rates when compared to those without mutations^20,36^. In our study, one-third of the patients who received ET with the CDK4/6 inhibitor experienced disease progression within 6 months after the start of treatments. Furthermore, the majority of these patients had polyclonal *ESR1* or *PIK3CA* mutations.

Although this study was planned to investigate prospectively the clinical usefulness of genetic analysis using cfDNA, it has several limitations. First, although the study was designed as a prospective cohort study, it remained an observational study without a direct intervention based on the mutations with which the patients presented. Second, a relatively small number of patients were involved, making it difficult to obtain statistically powerful results. Third, the dynamics of the clonal mutations were not observed because the majority of patients had not undergone a series of tests. Combining cfDNA mutation analysis with a serial follow-up for dynamics would be a more powerful predictive marker for ET with targeted therapies, especially targeting agents of the PI3K signaling pathway.

## Conclusions

In conclusion, *ESR1* and *PIK3CA* mutations in cfDNA were associated with clinical efficacies of endocrine therapy in HR+ MBC patients. Although the presence of *ESR1* or *PIK3CA* mutations was a poor predictive factor for endocrine therapy, patients with *PIK3CA* mutations were more likely to benefit from everolimus, mTOR inhibitor treatment. Based on our data, cfDNA analysis could be an applicable and useful guide for HR+ MBC patients who are candidates for endocrine therapy. However, further studies are needed, using a larger validation patient population, to confirm this possibility.

## Supplementary Information

Supplementary Figure S1.

Supplementary Table S1.

Supplementary Table S2.

Supplementary Table S3.

Supplementary Figure Caption.
