# Supplementary figures and images for: Mutation analysis using cell-free DNA for endocrine therapy in patients with HR+ metastatic breast cancer
Source: Sci Rep. 2021 Mar 10;11:5566. doi: 10.1038/s41598-021-84999-9 (PMC7946916; doi:10.1038/s41598-021-84999-9)

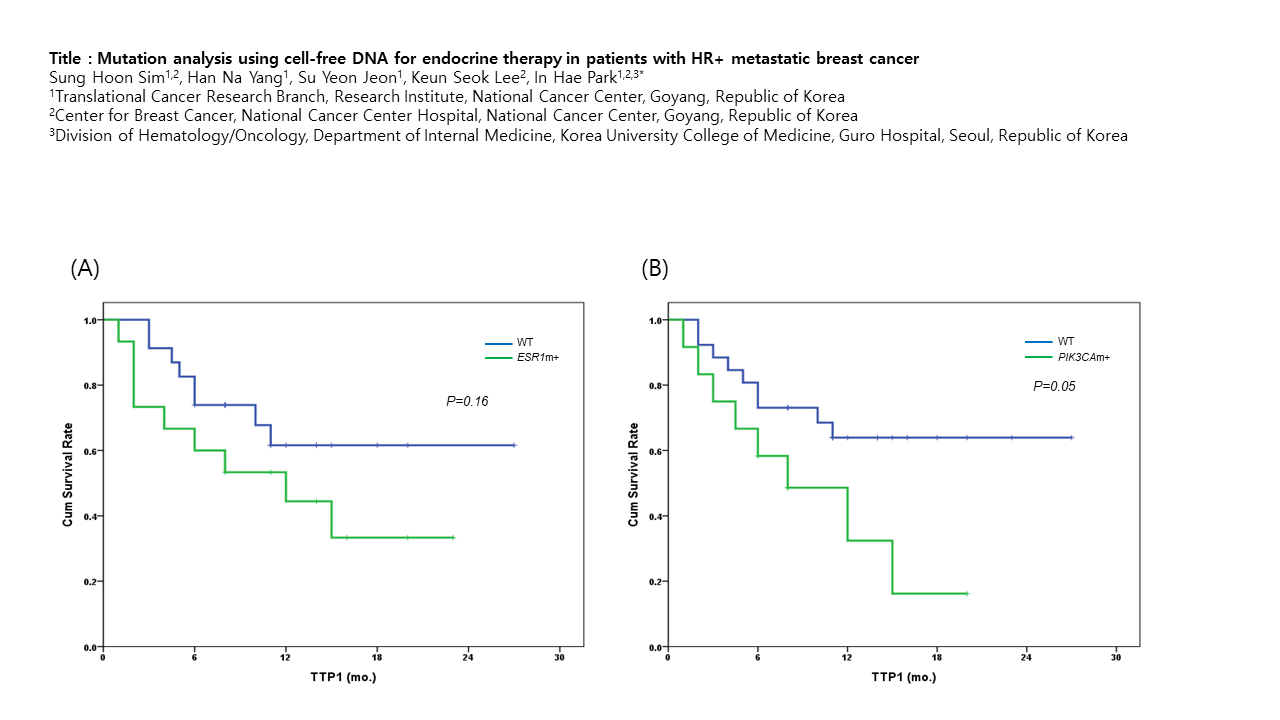

Supplement: Supplementary file 1 — Supplementary Figure S1. [file 41598_2021_84999_MOESM1_ESM.tif]
