# Supplementary material for: Mutation analysis using cell-free DNA for endocrine therapy in patients with HR+ metastatic breast cancer
Source: Sci Rep. 2021 Mar 10;11:5566. doi: 10.1038/s41598-021-84999-9 (PMC7946916; doi:10.1038/s41598-021-84999-9)
Supplement: Supplementary file 2 — Supplementary Table S1. [file 41598_2021_84999_MOESM2_ESM.docx]

**Title : Mutation analysis using cell-free DNA for endocrine therapy in patients with HR+ metastatic breast cancer**

Sung Hoon Sim^1,2^, Han Na Yang^1^, Su Yeon Jeon^1^, Keun Seok Lee^2^, In Hae Park^1,2,3*^

^1^Translational Cancer Research Branch, Research Institute, National Cancer Center, Goyang, Republic of Korea

^2^Center for Breast Cancer, National Cancer Center Hospital, National Cancer Center, Goyang, Republic of Korea

^3^Division of Hematology/Oncology, Department of Internal Medicine, Korea University College of Medicine, Guro Hospital, Seoul, Republic of Korea

**Table S1. Droplet digital (ddPCR) assay sequences for ESR1 mutation status**

| **ESR1 mutant** | **Forward Primer sequence** | **Reverse Primer Sequence** | **WT Probe Sequence** | **Mutant Probe Sequence** |
| --- | --- | --- | --- | --- |
| E380Q | GGATTTGACCCTCCATGA | AGACCAATCATCAGGATCTC | ACATTCTAGAAGGTGGACC | ACATTGTAGAAGGTGGACC |
| Y537S | GTACAGCATGAAGTGCAA | GGGCGTCCAGCATC | AGCAGGTCATAGAGGGG | AGCAGGTCAGAGAGGG |
| Y357N | GTACAGCATGAAGTGCAA | GGCGTCCAGCATCTC | CCCCTCTATGACCTGC | AGGTCATTGAGGGGCA |
| D538G | TACAGCATGAAGTGCAAG | TGGGCGTCCAGCA | CCCCTCTATGACCTGCT | TCTATGGCCTGCTGCT |
