# Supplementary material for: Mutation analysis using cell-free DNA for endocrine therapy in patients with HR+ metastatic breast cancer
Source: Sci Rep. 2021 Mar 10;11:5566. doi: 10.1038/s41598-021-84999-9 (PMC7946916; doi:10.1038/s41598-021-84999-9)
Supplement: Supplementary file 3 — Supplementary Table S2. [file 41598_2021_84999_MOESM3_ESM.docx]

**Title : Mutation analysis using cell-free DNA for endocrine therapy in patients with HR+ metastatic breast cancer**

Sung Hoon Sim^1,2^, Han Na Yang^1^, Su Yeon Jeon^1^, Keun Seok Lee^2^, In Hae Park^1,2,3*^

^1^Translational Cancer Research Branch, Research Institute, National Cancer Center, Goyang, Republic of Korea

^2^Center for Breast Cancer, National Cancer Center Hospital, National Cancer Center, Goyang, Republic of Korea

^3^Division of Hematology/Oncology, Department of Internal Medicine, Korea University College of Medicine, Guro Hospital, Seoul, Republic of Korea

**Table S2. The types of the first and second endocrine therapy (ET) after enrollment**

|  | **The first ET (n=75)** | **The second ET (n=12)** |
| --- | --- | --- |
| Letrozole+ palbociclib | 31 (41.3%) | 0 |
| Exemestane + everolimus | 19 (25.3%) | 9 (12.0%) |
| Fulvestrant + palbociclib | 7 (9.3%) | 2 (2.7%) |
| Letrozole | 9 (12.0%) | 0 |
| Fulvestrant | 8 (10.7%) | 1 (1.3%) |
| Tamoxifen | 1 (1.3%) | 0 |
