# Supplementary material for: Mutation analysis using cell-free DNA for endocrine therapy in patients with HR+ metastatic breast cancer
Source: Sci Rep. 2021 Mar 10;11:5566. doi: 10.1038/s41598-021-84999-9 (PMC7946916; doi:10.1038/s41598-021-84999-9)
Supplement: Supplementary file 4 — Supplementary Table S3. [file 41598_2021_84999_MOESM4_ESM.docx]

**Title : Mutation analysis using cell-free DNA for endocrine therapy in patients with HR+ metastatic breast cancer**

Sung Hoon Sim^1,2^, Han Na Yang^1^, Su Yeon Jeon^1^, Keun Seok Lee^2^, In Hae Park^1,2,3*^

^1^Translational Cancer Research Branch, Research Institute, National Cancer Center, Goyang, Republic of Korea

^2^Center for Breast Cancer, National Cancer Center Hospital, National Cancer Center, Goyang, Republic of Korea

^3^Division of Hematology/Oncology, Department of Internal Medicine, Korea University College of Medicine, Guro Hospital, Seoul, Republic of Korea

**Table S3. Cox analysis for time to progression of the first endocrine therapy after enrollment (TTP1) for all patient population**

|  | TTP1 | |
| --- | --- | --- |
|  | Univariable analysis | Multivariable analysis^*^ |
|  | 95% CI, P value | 95% CI, P value |
| Age (≥50 yrs vs. <50 yrs) | 1.0 (0.9-1.0), *p*=0.73 |  |
| Prior ET (yes vs. no) | 1.3 (1.0-1.9), *p*=0.09 | 1.0 (0.6-1.7), *p*=0.88 |
| Prior CT (yes vs. no) | 1.2 (1.0-1.4), *p*=0.05 | 1.1 (0.9-1.4), *p*=0.35 |
| Visceral metastasis (yes vs. no) | 0.9 (0.5-1.6), *p*=0.72 |  |
| *ESR1* m+ vs. WT | 1.8 (1.0-3.4), *p*=0.06 | 1.4 (0.7-2.9), *p*=0.41 |
| *PIK3CA* m+ vs. WT | 2.0 (1.0-3.8), *p*=0.04 | 1.7 (0.9-3.3), *p*=0.14 |

CI, confidential interval; ET, endocrine therapy; CT, chemotherapy; WT, wild type. *, adjusted with prior ET, prior CT, *ESR1*, and *PIK3CA* mutations.
