# Supplementary material for: Mutation analysis using cell-free DNA for endocrine therapy in patients with HR+ metastatic breast cancer
Source: Sci Rep. 2021 Mar 10;11:5566. doi: 10.1038/s41598-021-84999-9 (PMC7946916; doi:10.1038/s41598-021-84999-9)
Supplement: Supplementary file 5 — Supplementary Figure Caption. [file 41598_2021_84999_MOESM5_ESM.docx]

Figure S1. Time to progression for letrozole and palbociclib (CDK4/6 inhibitor) after enrollment (TTP1) according to the presence of *ESR1* (A) and *PIK3CA* mutations (B)
